# Enhancement of Closed-Loop Cognitive Stress Regulation Using Supervised Control Architectures

**DOI:** 10.1109/OJEMB.2022.3143686

**Published:** 2022-01-18

**Authors:** Hamid Fekri Azgomi, Rose T. Faghih

**Affiliations:** Department of Electrical and Computer EngineeringUniversity of Houston 14743 Houston TX 77004 USA; Department of Neurological SurgeryUniversity of California San Francisco 8785 San Francisco CA 94143 USA; Department of Biomedical EngineeringNew York University 5894 New York NY 10010 USA; Department of Electrical and Computer EngineeringUniversity of Houston 14743 Houston TX 77004 USA

**Keywords:** Closed-loop, cognitive stress, skin conductance, state-space, supervised control

## Abstract

*Goal:* We propose novel supervised control architectures to regulate the cognitive stress state and close the loop. *Methods:* We take information present in underlying neural impulses of skin conductance signals and employ model-based control techniques to close the loop in a state-space framework. For performance enhancement, we establish a supervised knowledge-based layer to update control system in real time. In the supervised architecture, the controller parameters are being updated in real-time. *Results:* Statistical analyses demonstrate the efficiency of supervised control architectures in improving the closed-loop results while maintaining stress levels within a desired range with more optimized control efforts. The model-based approaches would guarantee the control system-perspective criteria such as stability and optimality, and the proposed supervised knowledge-based layer would further enhance their efficiency. *Conclusion:* Outcomes in this in silico study verify the proficiency of the proposed supervised architectures to be implemented in the real world.

## I. Introduction

In The modern world, any challenge might be a source of cognitive stress [1]. The fast-paced life has the potential to induce emotional and cognitive stress [2]. Feeling overwhelmed, anxiety, and agitation are among the symptoms associated with the high levels of cognitive stress [3]. Conversely, loss of cognitive engagement might also prevent individuals from following their goals [4]. A low level of positive stress, which is also called eustress, might cause memory problems, lack of motivation, and poor concentration [5]. It can also negatively affect persons’ productivity in work places. While it is important to track internal stress levels [6], it is also critical to establish a mechanism for keeping internal cognitive stress state within a favorable range [7]. In this research, we aim to track the internal cognitive stress and propose novel control architectures to maintain it within the pleasant range. Advances in the fields of control and automation have opened avenues of applications in various area such as autonomous vehicles, robotics, and financial systems [8]. Recently, there has been much interest in investigating the use of modern control techniques in physiological systems [9]. Researchers are actively working on automating multiple clinical processes such as: artificial pancreas for regulating blood glucose levels [10], [11], feedback control mechanism in neuroprosthesis [12], internal energy regulation in patients with cortisol-related disorders [13]–[15], anesthesia delivery system for medically induced coma [16]–[18], and deep brain stimulation for treating neurodegenerative disorders [19]. Hence, we propose to employ control methods in internal cognitive stress regulation.

As internal cognitive stress state is a hidden state and can not be measured, we approach this problem indirectly [20]. In human body, the autonomic nervous system (ANS) is responsible for a vast number of functions in response to the mental stress [21]. Changes in the arousal of the sympathetic nervous system (SNS) and parasympathetic nervous systems (PSNS), as branches of ANS, are presented in different physiological signals[22]. In fact, the human brain employs SNS and PSNS to react to environmental stimuli. As a result of SNS and PSNS activation, we observe changes in physiological signals such as heart rate, respiration, and skin conductivity [23]. In response to internal/external stress stimuli, brain changes the sweat gland activation via SNS [24]–[26]. Consequently variations in sweat glands activation could be reflected in skin conductance signal monitored by sensors located in wrist-worn devices [27]. Skin conductance signal or electrodermal activity has been shown to be an indicator of mental arousal and cognitive stress [6], [28]–[30]. Therefore, we follow the approaches presented in [31] for further analysis. In the simulation system presented in [31], the hidden cognitive stress state is connected to the changes in skin conductance response (SCR) events via a state-space approach. Employing experimentally collected data, a real-time simulation system is developed to investigate the control design algorithm for closing the loop [31].

In the system presented in [7], [31], we took SCR events as the binary observation and estimated the hidden stress state in real time. While SCR time events carry important information about internal arousal state [6], [30], focusing on only the events’ time as the binary observations and ignoring their amplitudes may cause loss of valuable details. As reported in several articles [21], [29], SCR amplitudes includes information about internal arousal state. In [20], a modified version of the filtering approach, which incorporates continuous-valued information from the SCR amplitudes (i.e., phasic amplitude and tonic levels) is presented. In their proposed approach, they have reported overfitting to the continuous values [20]. To solve this issue, authors in [32] proposed the marked point process (MPP) filtering approach. The MPP filter is applied to estimate internal arousal state from SCR events and their corresponding amplitudes to address the overfitting problem [32]. Compared to our previous approach [31], which we only included SCR time events as binary observations, here we enhance the state estimation process by incorporating the event amplitudes and estimate the internal state with MPP approach.

Exploiting the state-space representation which will lead us to track internal arousal state in a systematic way, we aim to invest in control system techniques to regulate the estimated arousal state and close the loop. In recent years, there exists a growing interest in employing control methods to automate various procedures [33], [34]. Researchers in [35] developed a novel boundary control scheme to regulate a rigid-flexible wing system and close the loop. He *et al.* considered distributed disturbances and designed a robust control strategy to reject them [36]. Similarly, in present research, we propose novel control approaches to close the loop, regulate the estimated stress state, and keep it within the desired range. The state-space model and the real-time estimation enable us to handle this physiological system as a control-theoretic problem. Hence, we propose to employ well-established model-based optimal control techniques, including linear quadratic regulator (LQR) and model predictive control (MPC) to close the loop. In both LQR and MPC, by optimizing corresponding objective functions, the optimal control would be derived in a real-time manner. The performance of both LQR and MPC depends on the selection of the objective functions [37]. Additionally, due to the nature of this physiological system, the inter- and intra-subject variability make the objective function selection process a challenging task. Among available approaches that address the challenges associated with the objective function selection, research in [38] proposed to use genetic algorithm for optimal tuning of MPC weights. Ramasamy *et al.* have established a mechanism to update the cost functions based on the system performance as well as the operator input in an offline manner [38]. In their proposed approach, they use an interactive decision tree to get feedback from the operator and infer the optimal gain weights. Researchers in [39] proposed a multi-scenario approach for designing a robust MPC system. They evaluated the operational system for each scenario and considered them while tuning the MPC. Van *et al.* also proposed to combine the genetic algorithm with a multi objective fuzzy decision making system for MPC tuning [40]. In their proposed approach, they rank the predefined objective functions based on the fuzzy systems [40]. Zhao *et al.* in [41] implemented a real-time system for adjusting the MPC tuning parameters in an adaptive cruise control system. The expert system proposed in [41] adjusts the tune parameters based on if-then rules. The corresponding cost functions are regulated based on the changes in sign of error terms [41]. To address the need for creating a system to dynamically update the control tune parameters, we propose to establish a supervised layer on top of the implemented model-based control systems. In the proposed architectures, a knowledge-based fuzzy system would supervise the LQR and MPC and adjusts the objective functions in real-time.

The combination of fuzzy systems and model-based control techniques have been explored in the literature [42]–[44]. The researchers in [42] have used fuzzy logic methodology to address the output constraints while designing the MPC. Researchers in [43] use the fuzzy system to decouple the modeling process and use LQR approach to control the power plant. In a similar approach, researchers in [44] apply fuzzy system to model building heating system and implemented the MPC for the process control. However, the present work is the first attempt to use a fuzzy system as the supervised layer to adjust tuning parameters in model-based control structures. Moreover, the proposed supervised control architectures provide a setting to include the relevant medical expertise to enhance the closed-loop system. These novel supervised control approaches could be further expanded to deliver adaptive and robust closed-loop characteristics. The key contributions of the present research include (i) implementing real-time MPP Bayesian-type filter to estimate the hidden arousal state from amplitude and timings of skin conductance response events, (ii) taking advantage of state-space representation of internal arousal state and utilizing model-based LQR and MPC structures to regulate the hidden state, and (iii) presenting novel supervised fuzzy-LQR and fuzzy-MPC architectures to adjust control tuning parameters in real-time.

## II. Materials and Methods

**Fig. 1.**
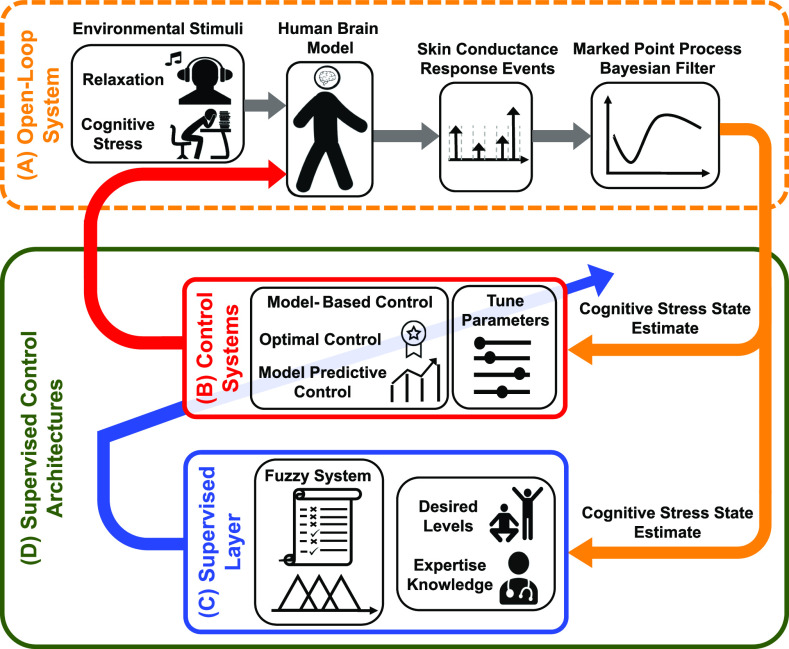
**Closed-Loop Supervised Control Architectures Overview.** (A) The orange dashed box displays the open-loop system. The solid green box, shows the supervised control architectures. (D) We take the SCR events generated by human brain model and utilize the MPP Bayesian filter to estimate the cognitive stress state. (B) To close the loop, we use the optimal control and model predictive control structures. (C) We establish a knowledge-based fuzzy system, as a supervised layer, and (D) apply expertise knowledge for updating the control tune parameters in a real-time manner.

An overview of the proposed closed-loop supervised control architectures is presented in Fig. 1. We utilize the simulation system presented in [31]. The idea presented in [31] is associated with employing experimental data [45] and simulating the environmental stimuli for two scenarios: cognitive stress and relaxation. In a state-space representation, we take simulated SCR events and estimate the hidden cognitive stress state in real-time. To this end, we employ the MPP Bayesian-type filtering ((A) in Fig. 1). To design the control signal and close the open-loop system, we use the model-based approaches LQR and MPC ((B) in Fig. 1). We establish a supervised fuzzy system on top of the LQR and MPC structures to automatically update the control tune parameters ((C) in Fig. 1). The supervised layer executes this task based on feedback from the estimated cognitive stress state, desired state levels, and expertise knowledge.

### A. Human Brain Stimulus-Response Model

We use the simulation model that is based on the experimental data [45] and presented in [31]. Non-EEG Dataset for Assessment of Neurological Status [45] is publicly available through the PhysioNet database [45], [46]. This study contains multiple experiments that induce different types of the stress to the subjects. The simulation model is based on two sessions: cognitive stress and relaxation, as the most representative cases [31]. In the original study [45], multiple physiological data were collected (i.e., skin conductance, body temperature, 3D accelerometer signals, heart rate, and blood oxygenation levels). In this research, we aim to track and regulate internal stress state by monitoring skin conductance measurements which were collected using Affectiva Q Curve wearable device to build the simulation environment. Similar to [6], [31], we analyze profiles associated with six selected participants whose data were clean and reliable. More information regarding this experiments and simulation system could be found in [6], [31], [45].

In the simulation system presented in [31], to model individual’s brain responses, we relate the internal cognitive stress-related state to the changes in skin conductance signal by employing a first-order state-space model [6], [30]:

\begin{equation*}
x_{k+1} = x_k + s_k + \nu _k + u_k \tag{1}
\end{equation*}

where $x_k$ stands for the hidden stress-related state, $ s_k$ reflects the environmental stimuli, and $\nu _k \sim \mathcal{N}\,\, (0,\,\sigma ^{2}_\nu)$ represents the process noise [20], [30]. $u_k$ denotes the control input signal designed and applied in real-time to regulate the simulated stress-related state. It is worth mentioning that we include the $s_k$ in (1) for the simulation purpose. In a real-world scenario, the human’s internal cognitive stress state is affected by real environmental stimuli. The details of modeling the environmental stimuli is presented in [31]. We also assume the occurrence of SCR events, $n_k$, follows a Bernoulli distribution with the following probability function:

\begin{equation*}
P(n_k | x_k)= q_k ^{n_k} (1- q_k)^{1-n_k} \tag{2}
\end{equation*}

where the probability $q_k$ is connected to the stress state $x_k$, via the following Sigmoid function [47]:

\begin{equation*}
q_k = \frac{1}{1+ e^{-(\gamma + x_k)}} \tag{3}
\end{equation*}

where $\gamma$ is the person-specific baseline parameter that should be determined. Similar to [31], we first assume $x_0$ approximately equals to zero. We then calculate the $\gamma$ based on the average probability of an SCR occurring in the whole data. According to (3), with increase in the levels of the cognitive stress state, the probability of receiving the SCR events is also increased.

To incorporate all the information included in SCR events, we extend our previous research [31], which only employs the SCR events’ time, to comprise the amplitudes associated with the SCR events. To this end, we assume there exists a linear relationship between the internal cognitive stress state $x_k$ and the SCR amplitudes:

\begin{equation*}
r_k = \rho _0 + \rho _1 x_k + \omega _k \tag{4}
\end{equation*}

where $r_k$ is assumed to be the log transformation of the continuous-valued associated with each SCR event’s amplitude. $\rho _0$ and $\rho _1$ are constant values derived by the offline expectation maximization algorithm [31], [32]. $\omega _k \sim \mathcal {N} (0,\sigma _\omega ^2)$ is measurement noise with variance $\sigma _\omega ^2$. Accordingly, the joint density function on the probability of receiving the SCR event $n_k$ with the corresponding amplitude $r_k$ is:

\begin{equation*}
p(n_k \cap r_k | x_k) = \left\lbrace \begin{array}{ll}q_k \frac{1}{\sqrt{2\pi \sigma _\omega ^2}}e^\frac{-(r_k -\rho _0 - \rho _1 x_k)^2}{2\sigma _\omega ^2} & \text{if } n_k =1, \\
 1 - q_k & \text{if } n_k = 0. \end{array}\right. \tag{5}
\end{equation*}

As presented in (5), the amplitude information will not be included when there is no impulse $(n_k = 0)$ [32].

It is worth mentioning that the log transformation, discussed in $r_k$ modeling (4), is only considered in this *in silico* study [20]. In real-world implementation of the proposed algorithm, we take amplitude and timing of SCR events to model and estimate cognitive arousal state [32].

### B. Cognitive Stress State Estimation via MPP Filtering

Taking the SCR events time and their corresponding amplitudes $(n_k,r_k)$, as the binary and continuous observations, we follow the MPP-based Bayesian filtering approach to estimate the hidden cognitive stress state $x_k$ [32]. While the estimation process includes the forward filter and a backward smoother, we only implement the forward part of the filter for further *real-time* analysis. At each time step, a Gaussian approximation is applied to the posterior density. Combining the prediction and the update steps in the forward filter [32], we estimate the stress state and its variance using the following recursive equations:

\begin{align*}
 \hat{x}_k =& \hat{x}_{k-1} + n_k C_k +(\hat{\sigma }^2_{k-1} + \sigma ^2_{\nu }) (n_k - q_k)\\
&\times \Bigg (\frac{(1 - n_k)\rho _1^2 (\hat{\sigma }^2_{k-1} +\sigma ^2_{\nu }) + \sigma ^2_{\omega }}{\rho _1^2 (\hat{\sigma }^2_{k-1} +\sigma ^2_{\nu }) + \sigma ^2_{\omega }} \Bigg) \tag{6}\\
\hat{\sigma }^2_{k} =& \left(\frac{1}{\hat{\sigma }^2_{k-1} + \sigma ^2_{\nu }} + q_k(1-q_k) + n_k D_k\right)^{-1} \tag{7}
\end{align*}

where,

\begin{equation*}
C_{k} = \frac{\rho _1 (\hat{\sigma }^2_{k-1} +\sigma ^2_{\nu })(r_k - \rho _0 - \rho _1 \hat{x}_{k-1})}{\rho _1^2 (\hat{\sigma }^2_{k-1} +\sigma ^2_{\nu }) + \sigma ^2_{\omega }}, D_{k} = \frac{\rho _1^2}{\sigma ^2_{\omega }} \tag{8}
\end{equation*}

when there exists a SCR event $(n_k \ne 0)$. Otherwise (i.e., $n_k = 0$), $C_k$ and $D_k$ equal zero $(C_k = D_k = 0)$. In fact, the terms $C_k$ and $D_k$ presented in (6) and (7) incorporate the continuous-valued information ($r_k$ in (4)) associated with the observed SCR event $n_k$ at time step $k$. So, these terms are applied only when there exists a SCR event $(n_k \ne 0)$. The probability $q_k$ presented in (6) and (7) is being related to the state $x_k$ via (3). So, it will results in a nonlinear problem that should be solved by employing numerical methods such as Newton-Raphson [34]. Consequently, we estimate the cognitive stress-related state $\hat{x}_k$ and its corresponding variance parameter $\hat{\sigma }_k$ in a real-time manner.

### C. Control Design

In this part, we follow the goal of establishing a knowledge-based fuzzy system ((C) in Fig. 1) as a supervised layer in model-based control approaches ((B) in Fig. 1) to close the loop and regulate the estimated cognitive stress state. Particularly, we implement the fuzzy control structure as a supervised layer in LQR and MPC structures. In the supervised architectures, the fuzzy system will automatically adjust the control tune parameters in real-time. In what follows, we discuss both model-based control approaches.

#### 1) LQR

Taking advantage of the state-space model and estimates of cognitive stress state, in LQR framework, we find the optimal solution of a predefined cost function. Hence, the obtained control signal $u_k$ will minimize the following objective function:

\begin{equation*}
J = \sum _{k=1}^K (\hat{x}_k - x_d)^{\prime }_k Q(\hat{x}_k -x_d) + u^{\prime }_k Ru_k \tag{9}
\end{equation*}

where $K$ is the ultimate time of the process. $Q$ and $R$ are positive definite weight matrices to penalize the state deviations and the input efforts, respectively. $x_d$ in (9) also stands for the desired levels of estimated stress state. Solving this optimization problem, the optimal control signal $u_k$ is derived as a linear state feedback controller:

\begin{equation*}
u_k = -G_k \hat{x}_k \tag{10}
\end{equation*}

where, the feedback gain $G_k$ is derived recursively:

\begin{equation*}
G_k = (R + P_{k+1})^{-1} P_{k+1} \tag{11}
\end{equation*}

where $P_k$ is the discrete solution of the following algebraic Riccati equation:

\begin{equation*}
P_k = Q + \left(P_{k+1} - P_{k+1}(R + P_{k+1})^{-1} P_{k+1}\right) \tag{12}
\end{equation*}

with the $P_K = Q$ initial condition.

#### 2) MPC

To advance the optimal control LQR, we propose to use MPC structure as the second model-based control technique. In MPC framework, we first project the state values for whole time-window horizon [48]. Then, we derive the control input for all future prediction window and apply the first control action. To this end, we introduce the following quadratic function that needs to be minimized:

\begin{equation*}
J_{{\mathbf u}_k} = \sum _{l=1}^{N_p} \hat{x}_{k+l|k}^{\prime } Q \hat{x}_{k+l|k} + \Delta u_{k+l|k}^{\prime } R \Delta u_{k+l|k} \tag{13}
\end{equation*}

where $N_p$ is the prediction horizon, $\hat{x}_{k+l|k}$ denotes to the state estimate prediction, and $\Delta u_{k+l|k} = u_{k+l+1|k} - u_{k+l|k}$ is the predicted variation of control input at each time step. Similar to LQR, $Q$ and $R$ are positive definite weight matrices to penalize the predicted state deviations and control efforts. To find the control signal, we aim to derive ${\mathbf u}_k = [u_{k|k} \; \; u_{k+1|k} \; \; \dots \; \; u_{k+N_p -1|k}]^{\prime }$ which is the control input for whole time horizon window prediction. To this end, we first define $\Delta \hat{x}_k = \hat{x}_{k} - \hat{x}_{k-1}$ and $\Delta u_k = u_{k} - u_{k-1}$. Using these terminologies, general state-space model (1) would be simply transferred to $\Delta \hat{x}_{k+1} = \Delta \hat{x}_{k} + \Delta u_{k}$. By considering the estimated state as the output equation, $y_k = \hat{x}_k$, and defining a new augmented variable, we build:

\begin{equation*}
x_a(k) = \left(\begin{array}{c}\Delta \hat{x}_k\\
 y_k \end{array}\right) \tag{14}
\end{equation*}

So, the augmented system dynamics would be such as:

\begin{align*}
x_a(k+1) &= A_a x_a(k) + B_a \Delta u(k) \tag{15}
\\
y(k)& = C_a x_a(k) \tag{16}
\end{align*}

where the augmented system matrices of (1) are:

\begin{equation*}
A_a = \left(\begin{array}{cc}1&0\\
1&1 \end{array}\right), \ B_a = \left(\begin{array}{c}1\\
1 \end{array}\right), \ C_a = \left(\begin{array}{cc}0&1 \end{array}\right) \tag{17}
\end{equation*}

Employing the output equation in the augmented system (16), we build the predicted future observation for whole prediction horizon $N_p$ such that:

\begin{equation*}
Y = W x_a(k) + Z\Delta U \tag{18}
\end{equation*}

where:

\begin{align*}
 Y =& \left(\begin{array}{c}y(k+1|k)\\
y(k+2|k)\\
\vdots \\
y(k+N_p|k) \end{array}\right), W = C_a \left(\begin{array}{c}A_a\\
A_a^2\\
\vdots \\
A_a^{N_p} \end{array}\right),\\
Z =& C_a \left(\begin{array}{cccc}B_a&&&\\
A_a B_a&B_a&&\\
\vdots &&\ddots &\\
A_a^{N_p-1} B_a&\dots &A_a B_a&B_a \end{array}\right) \tag{19}
\end{align*}

Now, the goal of finding control action $u_k$ is converted to calculating the sequence of $\Delta U = (\begin{array}{cc}\Delta u(k)&\Delta u(k+1)\ \cdots \ \Delta u(k+N_p -1)\end{array})$. Consequently, this sequence will provide the predicted state variables $ (\begin{array}{cc}x_a(k+1|k)&x_a(k+2|k)\ \cdots \ x_a(k+N_p|k) \end{array})$.

To find the sequence $\Delta U$ in (18), by knowing $Y,W,Z$ and $x_a(k)$, minimizing the cost function presented in (13) would be equal to minimizing the following objective function:

\begin{equation*}
J_{\Delta U} = Y^{\prime } Q_T Y + \Delta U^{\prime } R_T \Delta U \tag{20}
\end{equation*}

where $R_T= RI_{N_p \times N_p}$ and $Q_T= QI_{N_p \times N_p}$ are diagonal matrices for penalizing the control effort and deviations in the estimated state, respectively. Assuming there is no constraint, by setting $\frac{\partial J}{\partial \Delta U} = 0$, we derive the optimal solution:

\begin{equation*}
\Delta U^* = (R_T + Z^TQ_TZ)^{-1}Z^TQ_TWx_a \tag{21}
\end{equation*}

It is also worth mentioning that positive definite matrices $R_T$ and $Q_T$ (i.e., $R \succ 0$, $Q \succ 0$) will guarantee the second order necessary condition in the computed $\Delta U^*$. Finally, the first element in $\Delta U^*$, which is $\Delta u(k)$, includes required control action signal for each time step (i.e., $u_k = u_{k-1} + \Delta u(k)$).

It should be also noted that by any selections of positive definite weight matrices $Q$ and $R$, finding the optimal control would be equal to solving a quadratic program optimization problem (20). Solution $\Delta U^*$ in (21) only relies on the current state, past control input, and the desired level. Consequently, it will result in a closed-loop well-posed system that always has a unique solution [49].

In MPC design, while there exist methods for ensuring stability in infinite time horizon cases, utilizing a straightforward method for delivering rigorous stable property with finite time horizon remains challenging. In this research, to invest the stability, we evaluate prediction tail and consider terminal constraint [50]. Assuming terminal constraint $\hat{x}_{k+N_p} = x_d$ in (13) also provides with recursive feasibility. To this end, we consider the general form of optimal control input as Lyapunov function:

\begin{equation*}
V(k) = min \sum _{i=1}^{N_p} l(\hat{x}_k, \Delta u_k) \tag{22}
\end{equation*}

where $l(\hat{x}_k, \Delta u_k) = \hat{x}_k^{\prime } Q \hat{x}_k + \Delta u_k^{\prime } R \Delta u_k$. In $(k+1)$ time instant, the first component of $V(k+1)$ has been occurred and is no longer prediction. This unused part is called prediction tail (i.e., $ [\Delta u_{k+1} \; \; \cdots \; \; \Delta u_{k+N_p-1}]$) [50], [51]. For the sake of simplicity, we assume zero terminal constraint at this stage (i.e., $\hat{x}_{k+N_p} = 0$). Next, we follow the steps presented in [52] and derive $V(k+1)$:

\begin{equation*}
V(k+1) = V(k) - l(\hat{x}_{k}, \Delta u_0) + l(0, 0) \tag{23}
\end{equation*}

where initial cost $l(\hat{x}_{k}, \Delta u_0)$ is subtracted and corresponding cost for staying at terminal state is added (i.e, $l(0,0)$) [50]. Hence,

\begin{equation*}
V(k+1) - V(k) \leq - l(\hat{x}_{k}, \Delta u_0) \tag{24}
\end{equation*}

Since $l(\hat{x}_{k}, \Delta u_0) \geq 0$, we may conclude that $V(k+1) -V(k) \leq 0$ and the Lyapunov function candidate is stable.

### D. Supervised Control Architectures

As illustrated, in both LQR and MPC approaches, the selection of weight matrices $Q$ and $R$ plays an important role in the control design process. In fact, derived control gain in these model-based approaches highly depends on the weight matrices presented in (9) and (13). To update the weight matrices in real-time, we consider a knowledge-based system as a supervised layer in the design process. Therefore, we establish a fuzzy system on top of the pure LQR and MPC structures to (i) take the intrinsic advantages of the modeled dynamics employed in LQR and MPC, (ii) enhance the performance of the conventional architectures by adjusting the tune-parameters in real-time, and (iii) overcome the heuristic nature of the pure fuzzy control design (i.e., presented in [31]). To this end, we define the corresponding rule-base and fuzzy structure to change the tune-parameters (i.e., $Q$ and $R$ matrices) in real-time. On the basis of LQR and MPC, the larger $Q$ and $R$ values are, the more we penalize state deviations and control effort, respectively. Therefore, we set to use higher values for $Q$ while the error between the estimated state and target state levels is large and decrease it once the estimated stress state is within a predefined range. Following a similar logic, while the error term between the estimated state and the desired value is large, we set not to penalize the control input and let it minimize the error. Once the estimated state tends to a predefined range of the target level, we set to increase the $R$ and penalize the control effort to minimize it. Hence, we build the fuzzy rule base as presented in Table I.

**TABLE I:** Supervised Fuzzy Rule Base

Rule number	**IF**	**Then**	
	*e* error	*Q* parameter	*R* parameter
Rule$^1$	Large	Strong	Weak
Rule$^2$	Moderate	Moderate	Moderate
Rule$^3$	Small	Weak	Strong

**Fig. 2.**
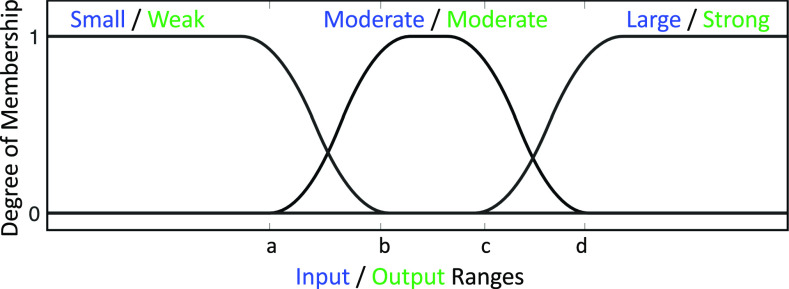
**Input and Output Membership Functions.** For each error input $e$ and the tune parameters $Q$ and $R$, three membership functions are employed to quantify the linguistic variables presented in Table I. Blue notations are for the error input and the green notations associated with the output tune parameters.

To quantify the linguistic variables presented in Table I, we employ the membership functions depicted in Fig. 2. According to the rule base (Table I), three sets of membership function for each input and output variables (i.e., error between the estimated state and the target level, $Q$ parameter, and $R$ parameter) are considered.

For each sets of input and outputs in Fig. 2, the middle functions belong to $\pi$-shaped membership functions with parameters $a,b,c$ and $d$. The left one and the right ones are z-shape function with the parameter $a$ and $b$ and s-shape function with parameters $c$ and $d$, respectively. To illustrate the shape of the membership function presented in Fig. 2, we present the middle $\pi$-functions as:

\begin{equation*}
\mu (x;a,b,c,d) = \left\lbrace \begin{array}{ll}0 & \text{if } x \leq a, \\
 2\left(\frac{x-a}{b-a}\right)^2 & \text{if } a \leq x \leq \frac{a+b}{2}, \\
 1- 2\left(\frac{x-b}{b-a}\right)^2 & \text{if } \frac{a+b}{2} \leq x \leq b, \\
 1 & \text{if } b \leq x \leq c, \\
 1- 2\left(\frac{x-c}{d-c}\right)^2 & \text{if } c \leq x \leq \frac{c+d}{2}, \\
 2\left(\frac{x-c}{d-c}\right)^2 & \text{if } \frac{c+d}{2} \leq x \leq d, \\
 0 & \text{if } x \geq d. \end{array}\right. \tag{25}
\end{equation*}

The s-shaped and z-shaped functions are spacial cases of the $\pi$-shaped function. The values associated with variables $a,b,c$ and $d$ for each input and output are presented in Table II. We also use the *Mamdani* inference engine and *centroid* defuzzification to execute the fuzzy system in the proposed supervised control architectures.

**TABLE II:** Membership Function Values in Supervised Layer (25)

Membership Function	Variable	a	b	c	d
Input **Error**	$e$ value	0.1	0.3	0.5	0.7
Output **Fuzzy-LQR**	$Q$ parameter	100	300	500	800
	$R$ parameter	5	20	30	45
Output **Fuzzy-MPC**	$Q$ parameter	1000	1500	2000	2500
	$R$ parameter	3	6	9	12

## III. Results

**Fig. 3.**
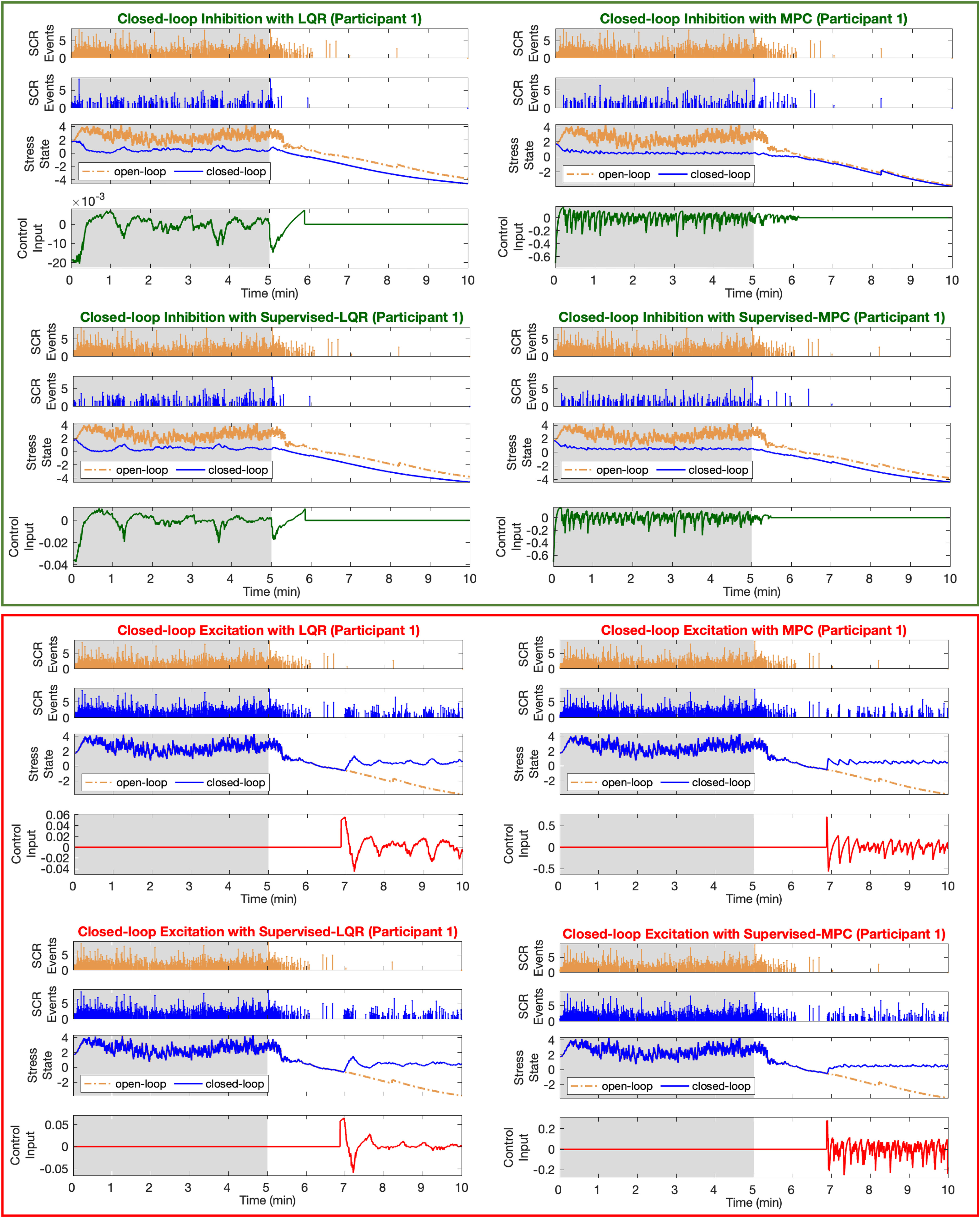
**Closed-Loop Results (Participant 1).** The top four panels show the closed-loop inhibition results. The bottom four panels show the closed-loop excitatory results. In each panel, the top two sub-panels show the SCR events along with their amplitudes in open-loop (orange color) and closed-loop (blue color) cases. The third sub-panel shows the estimated cognitive stress-related state. The bottom sub-panel shows the designed control implemented in real-time to close the loop and either inhibit or excite the estimated stress levels. The gray and white backgrounds correspond to the high and low arousal environmental stimuli, respectively (i.e., cognitive stress condition vs relaxing condition).

Implementing the model-based LQR and MPC methods in addition to the proposed supervised fuzzy-LQR and fuzzy-MPC approaches, we present the results. To show the performance of MPP filter in tracking cognitive stress state and demonstrate the efficiency of implementing the proposed supervised architectures, we present open-loop and closed-loop results. We follow the developed simulation environment in the order of first inducing cognitive stress and then causing the relaxation [31]. To analyze the accuracy of proposed control architectures, we present two closed-loop scenarios: inhibition for reducing the cognitive stress levels in the first half, and excitation to increase the levels of cognitive stress estimates in the second half of the simulation. In open-loop case, there is no control applied (i.e., $u_k=0$ in (1)). Fig. 3 depicts The results associated with the Participant 1. The results correspond to the rest of simulated profiles are presented in supplementary materials.

In each panel of Fig. 3, the top two sub-panels shows the simulated SCR events. The third sub-panel shows the estimated stress state. Orange and blue colors stand for open-loop and closed-loop results, respectively. The bottom sub-panels depict the resulted control signal (inhibitory control in green and excitatory control in red color).

### A. Closed-Loop Inhibition

The main goal in inhibitory closed-loop case is to design the control action to reduce the levels of the estimated cognitive stress state in the first half of the simulation. To investigate the effects of supervised layer, we present each model-based LQR and MPC methods along with their fuzzy supervised pairs (top four panels of Fig. 3). As presented in Fig. 3, control system detects high arousal levels and, by deriving the appropriate action, reduces the high levels of cognitive stress state in the first half of the simulation. As the second half is related to the low arousal period (or relaxation), there is no need to apply any control (i.e., $u = 0$). The left panels in Fig. 3 present the results of applying LQR and supervised fuzzy-LQR controllers. The right panels in Fig. 3 present the results of applying MPC and supervised fuzzy-MPC controllers.

### B. Closed-Loop Excitation

The main objective in excitatory closed-loop case is to design the control action for increasing the levels of the estimated cognitive stress state in the second half of the simulation (with low arousal environmental stimuli). The results of applying each model-based LQR and MPC method along with their fuzzy supervised pairs are presented in bottom four panels of Fig. 3. The excitatory control aims to detect the low levels of estimated cognitive stress state in the second half of the simulation and derive the appropriate control action to enhance it. As the first half is related to the high arousal (or cognitive stress stimuli), there is no need to apply any control action in this period (i.e., $u = 0$). The left panels in Fig. 3 present the results of applying LQR and supervised fuzzy-LQR controllers. The right panels in Fig. 3 present the results of applying MPC and supervised fuzzy-MPC controllers.

## IV. Discussion

In this research, as one of the very first in the context of closed-loop cognitive stress regulation, we proposed to use MPP filtering along with novel supervised control approaches to enhance the closed-loop control performance. In this regard, we utilized a simulation environment [31] based on the experimental data [45] to investigate the proposed methodologies in tracking and regulating internal cognitive stress state. To this end, we investigated skin conductance signal measurements and related them to the hidden stress state. To estimate the hidden state in real-time, we employed the MPP Bayesian-type filter and incorporated the information regarding the time and the amplitudes of SCR events.

The open-loop results, presented in Fig. 3, illustrate the sufficiency of internal stress state tracking in response to the changes in simulated environmental stimuli. The higher numbers/values of SCR events (i.e., orange spikes in the first sub-panel) and levels of estimated stress state (i.e. orange graph in the third sub-panel) in the first half of the simulation is because of the applied high arousal environmental stimuli. Moving toward the low arousal session (white background in Fig. 3), both the numbers/values of SCR events and the estimated stress levels drop significantly, which is due to the induced relaxing environmental stimuli in the second half of the simulation. These changes in the estimated stress state are in good agreement with the changes in SCR events: higher levels of the estimated stress state in the first half of the simulation (i.e., cognitive stress), and lower levels for the second half of the simulation (i.e., relaxation). These results verify the efficiency of the state-space approach along with the MPP filter in tracking the cognitive stress state in real-time.

To regulate the estimated stress levels in a closed-loop manner, we proposed novel supervised control approaches. Taking advantage of the state-space model as well as the real-time state estimation, we first presented the results of applying model-based system-theoretic control approaches: LQR and MPC. As the performance in these controllers highly depends on adjusting tune-parameters (i.e., weight matrices), we proposed a novel knowledge-based fuzzy supervised layer to enhance the control systems and update the control tuning parameters in real-time. The fuzzy system performs this task based on the insights into the system and changes in the control design criteria. The results of the proposed supervised control approaches in both inhibition and excitation cases are presented in Fig. 3.

In the closed-loop inhibition task (top four panels of Fig. 3), the goal is to reduce the levels of the estimated stress state in the stress session (i.e., first half of the simulation). During this period, we assume that the environmental stimuli cause the subject to feel stressed. As a result, SNS would activate the sweat glands and skin conductivity would be increased. Consequently, more activation on SCRs would be observed (top sub-panels of Fig. 3). By tracking the estimated stress state, the designed control system derives the required action for inhibition task. The control signal, presented in third sub-panel, is mainly active in the first half and results in lowering the stress state. The results of implementing supervised fuzzy-LQR approach is presented in the bottom left panel of Fig. 3. Establishing a supervised layer on top of the LQR approach results in achieving the control goal more precisely (second sup-panel) with more optimized control efforts. The results of applying MPC and supervised fuzzy-MPC approaches to inhibit the cognitive stress state are depicted in the right panels of Fig. 3. The control signal, presented in third sub-panel, is active in the first half of the simulation and tries to lower the estimated stress state. The results of implementing the supervised fuzzy system on top of the MPC system are presented in the bottom right panel of Fig. 3. This supervised architecture has improved the state tracking accuracy. Besides, the supervised layer has resulted in achieving the control goal with a more optimal control effort.

Compared to the inhibition task, the goal of implementing excitation class of controllers is to excite the low levels of arousal state. It is also important to keep the positive stress (i.e., eustress) in a desired range. The second half of the simulation in the presented environment is assumed to induce low cognitive stress condition on the person. We assume that the similar condition might happen while the subject is supposed to concentrate on the task, but due to multiple possible reasons, the cognitive engagement would be lost. The goal of elevating the estimated stress-related state has been followed by both designing the LQR and MPC approaches. The results of closed-loop excitation task are presented in the bottom four panels of Fig. 3.

First, by implementing the pure LQR method, the control action is active in the second half of the simulation, which is associated with the low arousal environmental stimuli. The LQR control action results in more activation in the simulated SCRs (first sub-panel), and leads to a higher level of estimated cognitive stress state (middle sub-panel). Enhancing the LQR closed-loop system by considering the supervised layer and updating the control tune-parameters in real-time, improves the results on both state tracking and control effort criteria. As presented in the bottom left panel of Fig. 3, the supervised fuzzy-LQR has led to a more precise state tracking with more optimal control efforts. As the second model-based approach, we implemented MPC method. First, by applying the pure MPC, the control action (third sub-panel) has elevated the levels of estimated stress state (second sub-panel). By enhancing the pure MPC structure with supervised fuzzy layer, we derive the results presented in the bottom right panel of Fig. 3. Similar to fuzzy-LQR, the supervised fuzzy-MPC architecture has improved the performance of the closed-loop excitation in both tracking accuracy and control effort minimization. To better evaluate the results of establishing supervised fuzzy system on top of model-based LQR and MPC approaches, we analyze the closed-loop results. Hence, we consider two criteria: (1) the effectiveness in reducing error term and improving the state tracking, and (2) achieving the closed-loop goal with optimized control efforts (see Table III).

**TABLE III:** Closed-Loop Performance Analysis (Participant 1)

Closed-Loop Class	Controller	$\frac{1}{K_{T}} \sum _{k=1}^{K_T} e_k^2$	$\frac{1}{K_{T}} \sum _{k=1}^{K_T} |u_k|$
Inhibition	LQR	0.1642	0.0045
	Supervised LQR	0.1260	0.0052
	MPC	0.0658	0.0662
	supervised MPC	0.0592	0.0590
Excitation	LQR	0.0664	0.0074
	supervised LQR	0.0640	0.0054
	MPC	0.0181	0.0621
	supervised MPC	0.0154	0.0338

In Table III, $e_k$ and $u_k$ represent the tracking error and the control input, respectively. $K_T$ is the total time that the control is active in the loop. As presented in Table III, the supervised layer in LQR structure has decreased the tracking error $e_k$ in inhibition task (0.1260 compared to 0.1642). Supervised fuzzy-LQR approach has improved state tracking accuracy by 23% with a 14% increase in the control efforts. In the excitation class, establishing supervised layer on top of the LQR system has resulted in a small improvement in state tracking accuracy (0.0640 compared to 0.0664) with a 27% decrease in total control efforts (0.0054 compared to 0.0074). Implementing the supervised fuzzy-MPC approach has resulted in more promising results. In comparison to the pure MPC, the supervised fuzzy-MPC system has reduced the tracking error by 10% and 15% in inhibition and excitation tasks, respectively. The supervised fuzzy-MPC architecture has also lead to applying less control efforts. It has reduced the total control effort by 10% and 45% in inhibition and excitation closed-loop tasks, respectively. The similar results for the rest of the simulated profiles are presented in the supplementary materials. We also analyzed the results of implementing supervised approaches on all six simulated profiles [6], [31]. A summary of overall closed-loop performance analysis for all simulated profiles are presented in Table IV.

**TABLE IV:** Overall Closed-Loop Performance Analysis

Closed-Loop Class	Criteria	Controller	Improvement
Inhibition	Average Error	Supervised LQR	+22.6%
		Supervised MPC	+23.0%
	Control Effort	Supervised LQR	-35.4%
		Supervised MPC	+7.6%
Excitation	Average Error	Supervised LQR	+5.4%
		Supervised MPC	+20.4%
	Control Effort	Supervised LQR	-0.0%
		Supervised MPC	+32.9%

As presented in Table IV, establishing supervised fuzzy system has significantly improved the MPC performance in both inhibition and excitation closed-loop systems. The proposed supervised fuzzy-MPC architecture has resulted in an enhanced tracking accuracy with more optimized control efforts. These analyses verify how the proposed supervised control architectures result in a more accurate state tracking with more optimal control efforts in MPC design. While the supervised fuzzy layer has also improved the tracking accuracy in LQR design, it has not been effective in accomplishing this task by reducing the control efforts. Supervised fuzzy-LQR system has decreased the tracking error on all six simulated profiles by average of 22.6% and 5.4% in inhibition and excitatory closed-loop classes, respectively. However, these improvements are not achieved by reducing the control efforts. Instead, in inhibition task, supervised LQR resulted in an average of 35% increase in control efforts. These analysis show that the proposed supervised architecture has great potential in improving state tracking accuracy in LQR design.

The results in this *in silico* study confirm that the proposed supervised architectures have great potentials to be implemented in real-world. The idea of applying a supervised layer on top of the model-based control approaches would result in performance improvement in closed-loop systems. It can also provide an excellent structure to incorporate medical expertise while designing the control. As we are dealing with a human-in-the-loop system, it is highly crucial to supervise the control systems. In the proposed supervised architectures, with respect to the nature of model-based LQR and MPC approaches, we ensure that the essential control system design criteria, such as stability and optimality, would be guaranteed. In fact, the supervised knowledge-based network would further enhance their efficiency by adjusting the control tune parameters in real-time. The proposed supervised methodologies are well-aligned to the human physiology basis and could be further investigated in similar closed-loop disorder treatments. These architectures could also be further expanded to result in adaptive and person-specific closed-loop tools.

## V. Conclusion

Influenced by the recent advances in wearable technologies and inspired by the fact that skin conductance carries important information regarding internal arousal state, we developed novel closed-loop architectures for regulating the hidden arousal state. To this end, we implemented marked point process filtering approach and included the amplitude and timing of skin conductance responses. To close the loop, we proposed supervised control techniques to take advantage of the state-space representation and model-based control methods. Hence, we established supervised LQR and supervised MPC structures for regulating the cognitive stress state. We investigated the efficiency of the proposed architecture in two class of closed-loop scenarios: inhibition and excitation. The results verify the effectiveness of proposed architectures in keeping the estimated stress state within a target range with more optimal control efforts.

## VI. Future Directions

As the next step of this research, we intend to investigate effects of possible safe actuation effective in regulating cognitive arousal state. By designing and performing human-subject experiments and modeling the actuation dynamics, we aim to include practical actuation while closing the loop. To this end, we can suggest to design different sets of experiments for inhibition and excitation purposes. For inhibition, one may consider designing the tasks that could increase individuals’ cognitive stress state. An example of these tasks is fear conditioning (e.g., watching the clips that may induce fear of heights in humans with acrophobia [53], [54]). While watching the clips, subjects should wear wearable devices that may collect their physiological data [55]. The goal of closing the loop would be incorporating the actuation to help them feel more relaxed. An example of real actuation in this example could be listening to relaxing music [56], [57] or performing diaphragmatic breathing [58]. For excitation purposes, one may design and perform experiments to help the subjects with enhancing their arousal state. In this regard, we can suggest to perform memory-related tasks [59] and analyze the effects of safe actuation helpful in elevating their arousal levels. In these experiments, subjects should fully engage with the tasks. The goal of closing the loop would be enhancing arousal state and improving cognitive performance. Therefore, one may measure performance state and further examine impacts of exciting actuation in both increasing arousal state and improving cognitive performance. Examples of safe exciting actuation could be drinking beverages like coffee or energy drinks [60], [61]. Employing experimental data in these closed-loop experiments, one may perform system identification to model these safe actuation’s dynamics. Once we learn how a specific actuation would affect one’s arousal state, we may incorporate their dynamics while closing the loop.

In the aforementioned experiments, we expect to observe variable responses among different subjects. Moreover, as uncertainty in model parameters presented in the state-space representation is unavoidable, it is also beneficial to research on adaptive and robust control design to further enhance the control systems [62]. Another future direction of this research could be extending the proposed architectures and considering adaptive and robust properties in both the state estimation and control design stages. The perspective closed-loop systems would be adaptive to the uncertainty in the modeled dynamics and robust to unexpected disturbances. Consequently, the idea of closed-loop cognitive stress regulation would be applicable in real-world situations. In an actual environment, a wrist-worn device would collect the skin conductance signal. Utilizing the proposed methods, the internal arousal state would be estimated in real-time. Taking advantage of the proposed experiments, one may incorporate actuation dynamics while implementing supervised control architectures for closing the loop regulating the arousal state.

## Supplementary Materials

Supplementary materials.

A detailed description of the methodology, including (I) Instrumentation, (II) Data Processing, (III) Breathing Patterns, and (IV) Cough Generator.
