# Supplementary material for: Enhancement of Closed-Loop Cognitive Stress Regulation Using Supervised Control Architectures
Source: IEEE Open J Eng Med Biol. 2022 Jan 18;3:7–17. doi: 10.1109/OJEMB.2022.3143686 (PMC8979622; doi:10.1109/OJEMB.2022.3143686)
Supplement: A detailed description of the methodology, including (I) Instrumentation, (II) Data Processing, (III) Breathing Patterns, and (IV) Cough Generator. [file supp1-3143686.pdf]

Supplementary Materials

Enhancement of Closed-Loop Cognitive Stress Regulation using Supervised Control Architectures

Hamid Fekri Azgomi, *Student Member, IEEE,* Rose T. Faghih*, *Senior Member, IEEE*

THE closed-loop results for the rest of the simulated profiles are presented in Figs. 1–5. The closed-loop analysis for the rest of the simulated profiles are also presented in Tables I–V.

TABLE I. Closed-Loop Performance Analysis (Participant 2).

Closed-Loop Class	Controller	$\frac{1}{K_T}\sum_{k=1}^{K_T} e_k^2$	$\frac{1}{K_T}\sum_{k=1}^{K_T} \lvert u_k \rvert$
Inhibition	LQR	0.0630	0.0038
	Supervised LQR	0.0434	0.0047
	MPC	0.0394	0.0415
	Supervised MPC	0.0261	0.0402
Excitation	LQR	0.0744	0.0047
	Supervised LQR	0.0599	0.0051
	MPC	0.0123	0.0444
	Supervised MPC	0.0149	0.0294

TABLE II. Closed-Loop Performance Analysis (Participant 3).

Closed-Loop Class	Controller	$\frac{1}{K_T}\sum_{k=1}^{K_T} e_k^2$	$\frac{1}{K_T}\sum_{k=1}^{K_T} \lvert u_k \rvert$
Inhibition	LQR	0.2356	0.0076
	Supervised LQR	0.1841	0.0138
	MPC	0.1059	0.1385
	Supervised MPC	0.0611	0.1249
Excitation	LQR	0.1416	0.0107
	Supervised LQR	0.1381	0.0113
	MPC	0.0580	0.1489
	Supervised MPC	0.0372	0.0931

TABLE III. Closed-Loop Performance Analysis (Participant 4).

Closed-Loop Class	Controller	$\frac{1}{K_T}\sum_{k=1}^{K_T} e_k^2$	$\frac{1}{K_T}\sum_{k=1}^{K_T} \lvert u_k \rvert$
Inhibition	LQR	0.1429	0.0059
	Supervised LQR	0.1119	0.0102
	MPC	0.3438	0.1390
	Supervised MPC	0.2780	0.1235
Excitation	LQR	0.0967	0.0115
	Supervised LQR	0.0954	0.0085
	MPC	0.0736	0.1699
	Supervised MPC	0.0494	0.1309

TABLE IV. Closed-Loop Performance Analysis (Participant 5).

Closed-Loop Class	Controller	$\frac{1}{K_T}\sum_{k=1}^{K_T} e_k^2$	$\frac{1}{K_T}\sum_{k=1}^{K_T} \lvert u_k \rvert$
Inhibition	LQR	0.1584	0.0093
	Supervised LQR	0.1225	0.0107
	MPC	0.1110	0.1214
	Supervised MPC	0.0859	0.1093
Excitation	LQR	0.0823	0.0151
	Supervised LQR	0.0868	0.0081
	MPC	0.0348	0.1162
	Supervised MPC	0.0221	0.0810

TABLE V. Closed-Loop Performance Analysis (Participant 6).

Closed-Loop Class	Controller	$\frac{1}{K_T}\sum_{k=1}^{K_T} e_k^2$	$\frac{1}{K_T}\sum_{k=1}^{K_T} \lvert u_k \rvert$
Inhibition	LQR	0.2751	0.0145
	Supervised LQR	0.2334	0.0154
	MPC	0.3458	0.1401
	Supervised MPC	0.3087	0.1391
Excitation	LQR	0.1542	0.0058
	Supervised LQR	0.1261	0.0108
	MPC	0.0471	0.1330
	Supervised MPC	0.0362	0.0961

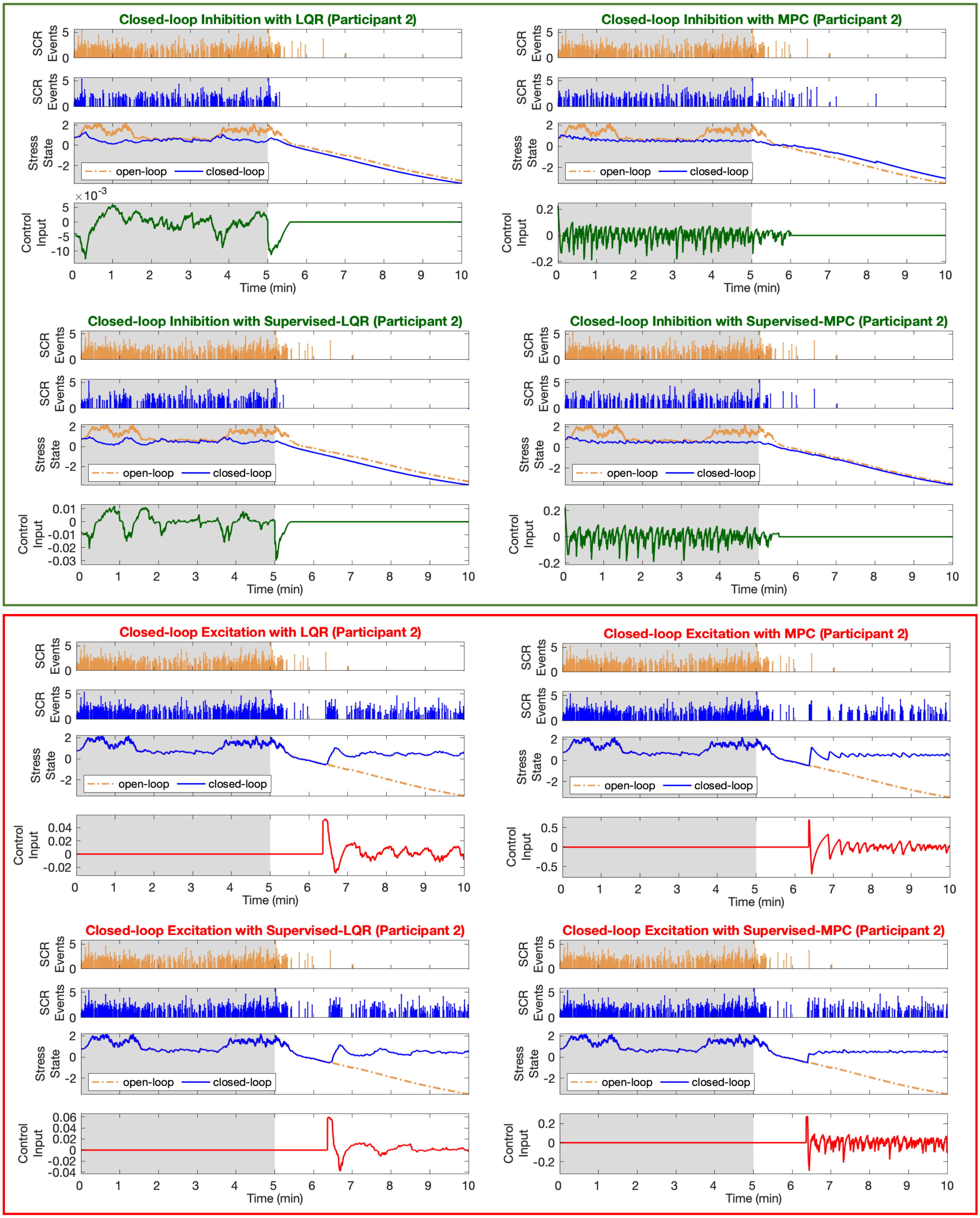

Fig. 1. **Closed-Loop Results (Participant 2).** The top four panels show the closed-loop inhibition results. The bottom four panels show the closed-loop excitatory results. In each panel, the top two sub-panels show the SCR events along with their amplitudes in open-loop (orange color) and closed-loop (blue color) cases. The third sub-panel shows the estimated cognitive stress-related state. The bottom sub-panel shows the designed control implemented in real-time to close the loop and either inhibit or excite the estimated stress levels. The grey and white backgrounds correspond to the high and low arousal environmental stimuli, respectively (i.e., cognitive stress condition vs relaxing condition).

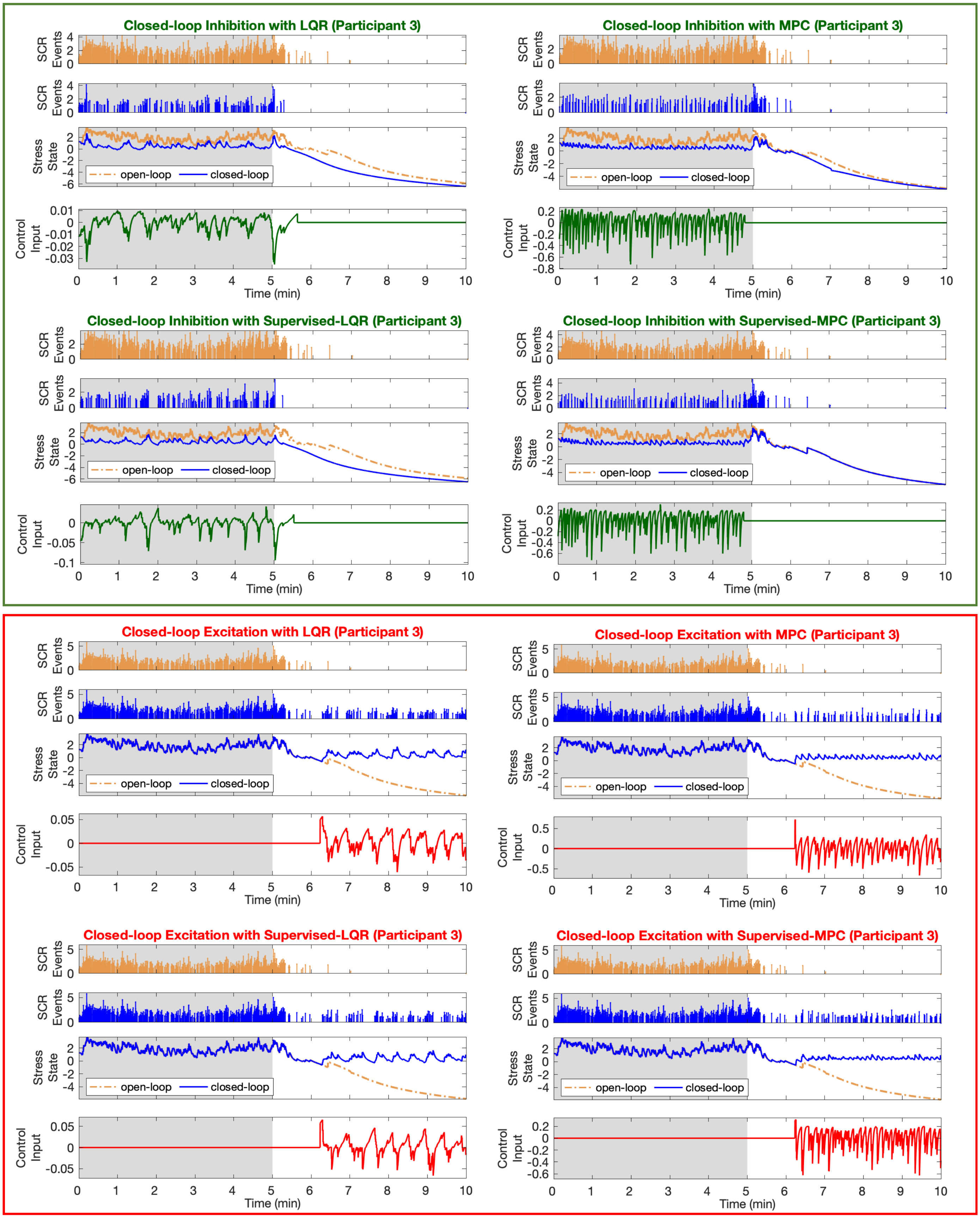

Fig. 2. **Closed-Loop Results (Participant 3).** The top four panels show the closed-loop inhibition results. The bottom four panels show the closed-loop excitatory results. In each panel, the top two sub-panels show the SCR events along with their amplitudes in open-loop (orange color) and closed-loop (blue color) cases. The third sub-panel shows the estimated cognitive stress-related state. The bottom sub-panel shows the designed control implemented in real-time to close the loop and either inhibit or excite the estimated stress levels. The grey and white backgrounds correspond to the high and low arousal environmental stimuli, respectively (i.e., cognitive stress condition vs relaxing condition).

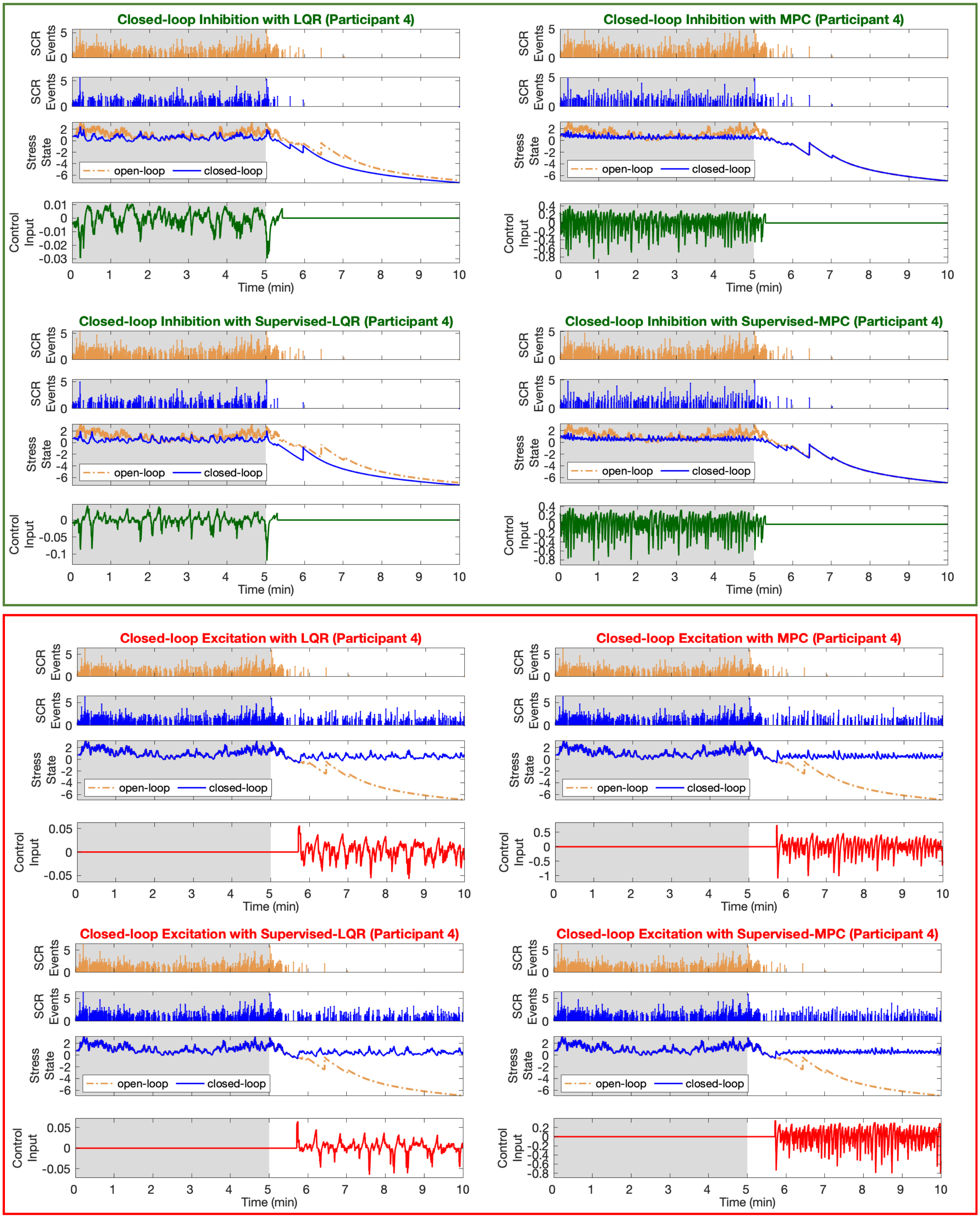

Fig. 3. **Closed-Loop Results (Participant 4).** The top four panels show the closed-loop inhibition results. The bottom four panels show the closed-loop excitatory results. In each panel, the top two sub-panels show the SCR events along with their amplitudes in open-loop (orange color) and closed-loop (blue color) cases. The third sub-panel shows the estimated cognitive stress-related state. The bottom sub-panel shows the designed control implemented in real-time to close the loop and either inhibit or excite the estimated stress levels. The grey and white backgrounds correspond to the high and low arousal environmental stimuli, respectively (i.e., cognitive stress condition vs relaxing condition).

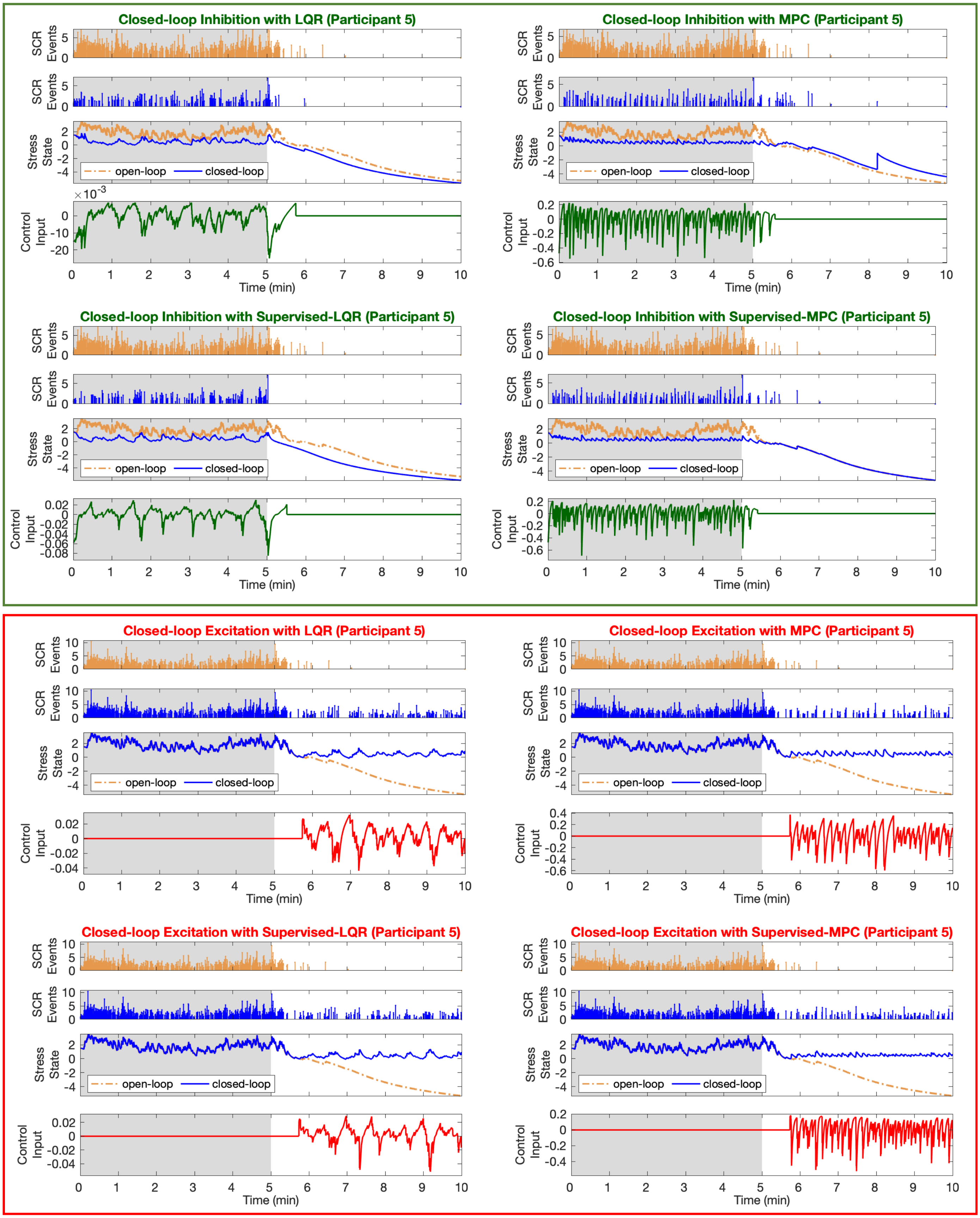

Fig. 4. **Closed-Loop Results (Participant 5).** The top four panels show the closed-loop inhibition results. The bottom four panels show the closed-loop excitatory results. In each panel, the top two sub-panels show the SCR events along with their amplitudes in open-loop (orange color) and closed-loop (blue color) cases. The third sub-panel shows the estimated cognitive stress-related state. The bottom sub-panel shows the designed control implemented in real-time to close the loop and either inhibit or excite the estimated stress levels. The grey and white backgrounds correspond to the high and low arousal environmental stimuli, respectively (i.e., cognitive stress condition vs relaxing condition).

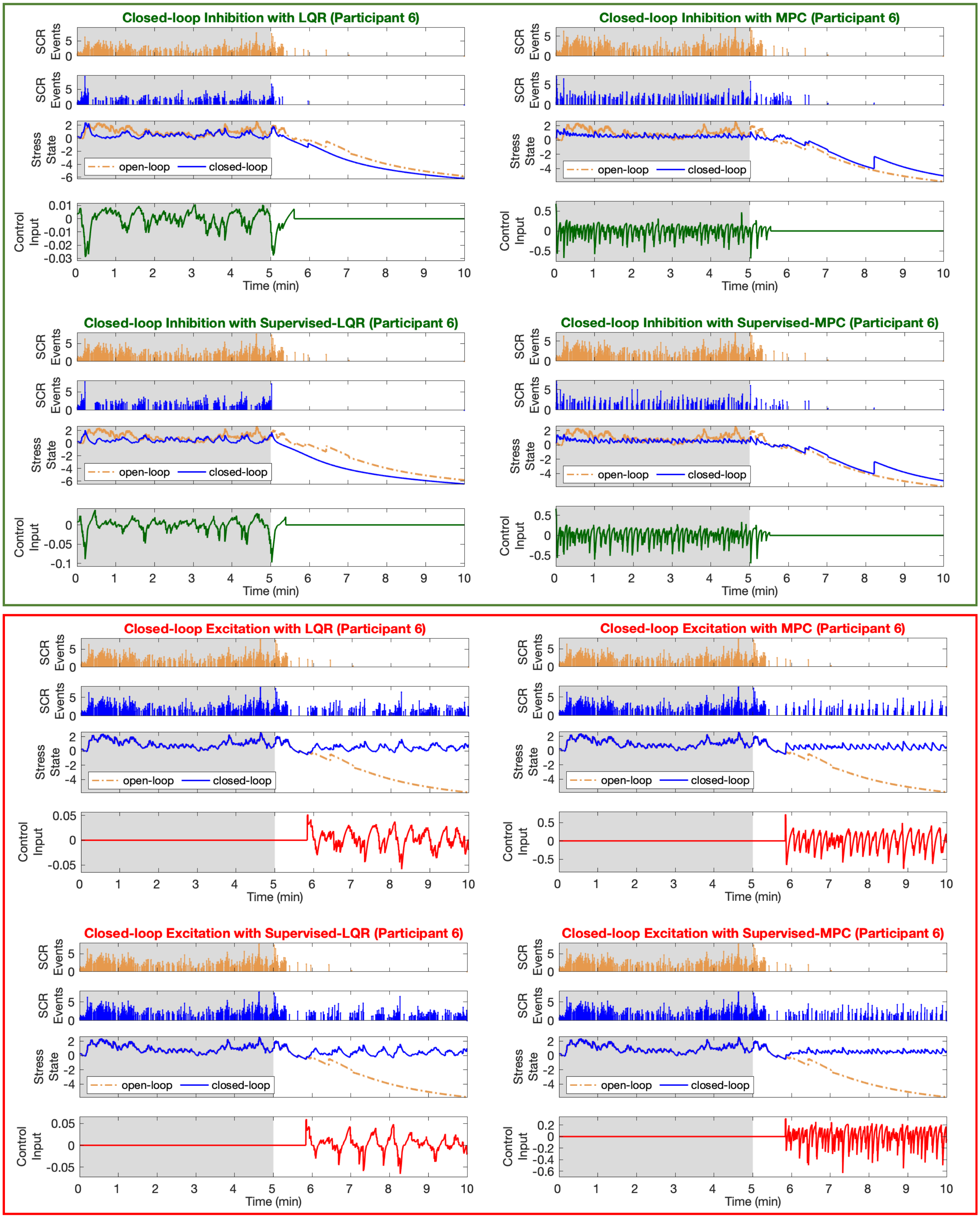

Fig. 5. **Closed-Loop Results (Participant 6).** The top four panels show the closed-loop inhibition results. The bottom four panels show the closed-loop excitatory results. In each panel, the top two sub-panels show the SCR events along with their amplitudes in open-loop (orange color) and closed-loop (blue color) cases. The third sub-panel shows the estimated cognitive stress-related state. The bottom sub-panel shows the designed control implemented in real-time to close the loop and either inhibit or excite the estimated stress levels. The grey and white backgrounds correspond to the high and low arousal environmental stimuli, respectively (i.e., cognitive stress condition vs relaxing condition).
